# Best humans still outperform artificial intelligence in a creative divergent thinking task

**DOI:** 10.1038/s41598-023-40858-3

**Published:** 2023-09-14

**Authors:** Mika Koivisto, Simone Grassini

**Affiliations:** 1 Department of Psychology, University of Turku, Turku, Finland; 2 Department of Psychosocial Science, University of Bergen, Bergen, Norway; 3 Cognitive and Behavioral Neuroscience Laboratory, University of Stavanger, Stavanger, Norway

**Keywords:** Psychology, Mathematics and computing

## Abstract

Creativity has traditionally been considered an ability exclusive to human beings. However, the rapid development of artificial intelligence (AI) has resulted in generative AI chatbots that can produce high-quality artworks, raising questions about the differences between human and machine creativity. In this study, we compared the creativity of humans (n = 256) with that of three current AI chatbots using the alternate uses task (AUT), which is the most used divergent thinking task. Participants were asked to generate uncommon and creative uses for everyday objects. On average, the AI chatbots outperformed human participants. While human responses included poor-quality ideas, the chatbots generally produced more creative responses. However, the best human ideas still matched or exceed those of the chatbots. While this study highlights the potential of AI as a tool to enhance creativity, it also underscores the unique and complex nature of human creativity that may be difficult to fully replicate or surpass with AI technology. The study provides insights into the relationship between human and machine creativity, which is related to important questions about the future of creative work in the age of AI.

## Introduction

The development and widespread availability of generative artificial intelligence (AI) tools, such as ChatGPT (https://openai.com/) or MidJourney (https://www.midjourney.com), has sparked a lively debate about numerous aspects of their integration into society^1^, as well as about the nature of creativity in humans and AI^2^. One of the key issues surrounding the implementation of AI technologies pertains to their potential impact on the job market^3^. With AI systems becoming increasingly capable of performing tasks that were once solely within the purview of humans, concerns have been raised about the potential displacement of jobs and its implications for future employment prospects^4^. In the field of education, questions have been raised about the ethical and pedagogical implications of such technologies, as well as concerns about how AI systems might reduce critical thinking skills^5^. Another aspect of the debate involves the legal and ethical ramifications of AI-generated content^6,7^. As these tools produce increasingly sophisticated works, ranging from articles to artistic creations, it raises the issue of whether AI-generated products should be granted the same legal protections as human-created works, and how to assign responsibility and credit for such creations.

At the heart of these discussions lie fundamental questions about the nature of human identity and creativity, and how this identity interfaces with AI systems that seem capable of human-like creative production^2^. As AI technologies continue to advance, they challenge traditional notions of what it means to be human and force us to reconsider the unique qualities that define our species. For example, the concept of creativity, which has historically been attributed exclusively to conscious human beings^8,9^, is now being reevaluated considering AI's ability to seemingly generate original content.

AI has shown tremendous potential for greater and more enormous possibilities in areas that require reasoning and creative decision making. This is demonstrated, for example, by the rise of chess engines, neural networks, and deep learning-based chess networks, which are capable of defeating chess masters (https://builtin.com/artificial-intelligence/chess-ai). Additionally, AI seems to perform well in art-related creativity. Recent AI tools can produce high-quality art pieces that have been bought for high prices^10^, as well as poetry that is indistinguishable from human-made art^11^. These findings seem to suggest that AI is capable of creating products that humans typically perceive as creative. But what exactly is creativity?

Traditionally, creativity has been defined as the ability to produce ideas that are, to some extent, both original and useful^12^. This definition allows us to evaluate the creativity of AI's ideas using the same criteria applied to human ideas. In this study, we compare the products generated by AI and humans in the context of creative thinking. Guilford^13^ distinguished between convergent and divergent thinking. Convergent thinking refers to the ability to determine the single best or correct answer to a problem, whereas divergent thinking involves generating many different ideas or solutions. Divergent thinking, more so than convergent thinking, has been closely associated with creativity and the ability to envision numerous potential answers to a question. Divergent thinking can be further divided into specific components: fluency (the ability to produce a large number of ideas), flexibility (the ability to think about a topic from different perspectives), originality (the ability to produce unique or novel ideas), and elaboration (the ability to expand upon or add detail to ideas). However, the assumption that divergent thinking would in fact represent creativity as a phenomenon has been disputed^14^. Nevertheless, divergent thinking has often been measured in the context of psychological research on creativity and tasks measuring divergent thinking are well established^15^.

The most accepted theories regarding the creative process are based on the dual-process view. Guilford's^16^ model proposed that the creative process involves an interplay between spontaneous (divergent) and controlled (convergent) modes of thinking. The more spontaneous divergent thinking is responsible for the originality and novelty of the ideas, whereas the controlled process evaluates the relevance of the ideas in relation to the demands of the task. The *associative theory* of creativity^17,18^ assumes that creative ideas result from making connections between weakly related concepts to form novel ideas. This theory proposes that individuals with a flat structure of semantic knowledge are more likely to activate and associate remote ideas, thus increasing the probability of forming original combinations of ideas compared to those with strictly hierarchical or steep structures. This view is supported by recent computational methods^19^ and functional brain imaging studies^20^, which suggest that creative individuals have more connected and flexible semantic networks than less creative individuals.

The controlled-attention theory emphasizes that executive functions are necessary for creative idea generation^21,22^. These accounts can be integrated into a hybrid view assuming that bottom-up, associative processes are beneficial for creative thinking, while top-down processes contribute by providing executive control during the retrieval of concepts from semantic memory. For example, top-down processes during creative process can generate and maintain retrieval cues, inhibit salient and highly associated information, and shift attention^21,22^.

Divergent thinking has traditionally been assessed by tests requiring open-ended responses^16^. The most used test of divergent thinking is the Alternate Uses Task (AUT), in which participants are asked to produce uncommon, creative uses for everyday objects (e.g., brick). We investigated the differences in creative potential between humans and AI chatbots using the AUT. The key process of creative thinking in humans is the ability to access remotely related concepts. Current AI chatbots have a vast memory and the ability to quickly access large databases. Therefore, one might hypothesize that AI chatbots will outperform humans in the associative component of divergent thinking, and thus in the originality of responses. To operationalize originality, we used a computational method^23^ to objectively quantify the semantic distance between the object probes and the responses. Additionally, human raters who were blind to the presence of AI-generated responses evaluated the responses. These raters provided a human view of creativity, as it is possible that mere semantic distance may not capture all aspects of creative products that humans consider original or surprising.

## Method

### Participants

The AUT data from human participants were collected in the context of another study and its’ method was preregistered at OSF.io in the context of that study (https://osf.io/fy3mn/?view_only=f1cf960d0170433dba9d31df68a6eaf7). Native English speakers were recruited via the online platform Prolific (www.prolific.co) and paid 2£ for the about 13-min participation. A total of 310 participants opened the link to the study and full data was obtained from 279 participants who performed the study from the start to the end. Of the 279 participants with full data, 256 passed the attention checks (108 females, 145 males, 2 other, and 1 preferred not to identify the gender identity) and their results were included in the present study. The attention checks consisted of easy visual detection and recognition tasks. The average age of the participants was 30.4 years, ranging from 19 to 40 years; 44 of them were students. The employment status was fulltime for 142, part time for 37, unemployed for 30, and other, homemaker, retired or disabled for 42. The participants reported no head injury, medication, or ongoing mental health problems. They resided in the United Kingdom (n = 166), USA (n = 79), Canada (n = 9), or Ireland (n = 2). All participants provided informed consent prior to the start of the study. The collection of the human data was performed in accordance with the Declaration of Helsinki and it had the acceptance of Ethics Committee for Human Sciences at the University of Turku.

The AI chatbots ChatGPT3.5 (referred as ChatGPT3 in the following text) ChatGPT4, and Copy.Ai (based on the GPT 3 technology) were tested. ChatGPT3 was tested 30.3.2023, ChatGPT4 was tested 5.4.2023, and Copy.Ai was tested 1.4.- 2.4.2023. Each chatbot was tested 11 times with four object prompts in different sessions. We did not want to increase the number of sessions as we noted during piloting that the chatbots tended to repeat some responses across the sessions, although the combination of the responses to each object was different between the sessions. Thus, we had 11 test sessions with the four objects for each chatbot (n = 132 observations). This seemed a reasonably large sample to obtain sufficient power to detect differences at 0.05 alpha level when compared to the 256 humans’ 1024 observations in single trial analyses.

### Procedure

The Alternate Uses Task (AUT) included four tasks with the object probes *rope, box, pencil,* and *candle*, respectively. Before staring the tasks, the human participants were presented with the instruction stressing quality instead of quantity, following the guidelines given by Beaty and Johnson^23^: “*For the next task, you'll be asked to come up with original and creative uses for an object. The goal is to come up with creative ideas, which are ideas that strike people as clever, unusual, interesting, uncommon, humorous, innovative, or different. Your ideas don't have to be practical or realistic; they can be silly or strange, even, so long as they are creative uses rather than ordinary uses. You may type in as many ideas as you can, but creative quality is more important than quantity. It's better to have a few really good ideas than a lot of uncreative ones. You have 30 s to respond each object*”. After having read the instruction, the tasks started. Each object name was presented for 30 s, during which the participants entered their ideas into text boxes located below the object name. In the beginning of each task, the participants were reminded that they should “*come up original and creative uses for an object*”.

Although each human was tested once with the four objects in one session, the testing of each AI chatbot consisted of 11 sessions with each object. The four objects were tested always once within one session (i.e., chat/conversation), after which the session was closed, and a new session was started so that the memory of AI was cleared from the contents of the previous session. The instructions for AI were otherwise identical to those given for humans, but two exceptions had to be made. First, piloting with the chatbots suggested that if ChatGPT3 were given no explicit restriction to the number of ideas, it always generated 10 ideas, while ChatGPT4 generated between 7 and 8 ideas; Copy.Ai generated a more variable number of ideas. To restrict the number of ideas so that they would correspond to those given by humans, we first examined the distribution of the number of human ideas. The median number and mode for humans was 3 ideas, with slightly rightward tail in the distribution (see Supplementary Materials, Supplementary Fig. 1). Therefore, we asked the AIs 4 times to generate 3 ideas, 2 times 2 ideas, 2 times 4 ideas, and once to generate 1, 5 and 6 ideas, to control for the number of ideas (i.e., fluency). Thus, the sentence “you can type in as many ideas as you can” in the humans’ instruction was changed to “*You can type in one [or two/three/four/five/six] ideas*.” In addition, without any restriction in the number of words for expressing the ideas, the AIs would have generated rather long and elaborated responses, which are not comparable to the human responses which consisted typically of 1 – 3 words (e.g., “cat playhouse” in response to *box*). Therefore, we added to the end of the instruction: “*Use only 1–3 words in each response*.” ChatGPT3 and ChatGPT4 followed well the instructions, while Copy.Ai sometimes needed further instructions, for example such as “*I asked for three ideas*”, or “*State your previous response with 1 – 3 words*.”

### Scoring

The responses were spell-checked before entering them into analyses. If the response contained only few letters and it was not clear what the participant had meant, the response was removed from the semantic distance analysis. For this reason, or due to participants inability to produce any response to a particular object within 30 s, 14 participants’ data is not fully complete, but lacks observation in some of the cells corresponding to the specific object (this explains also why the degrees of freedom may vary in the statistical analyses).

The originality of divergent thinking was operationalized as semantic distance between the object name and the AUT response. The semantic distance was determined with SemDis platform (semdis.wlu.psu.edu; ^23^). In the semantic distance analysis, “multiplicative” compositional model option in SemDis was used to account for AUT responses with multiple words. The responses were preprocessed using the “remove filler and clean” setting which removes “stop words” (e.g., *the, an, a, to*) and punctuation marks that can confound semantic distance computation. In addition, other editing of the responses was needed to control the confounding effects between humans and AIs. The AIs used relatively often the expression “DIY” (“do it yourself”): GPT3 four times, Copy.Ai seven times, and GTP4 three times, whereas the 256 humans used it only a total of two times. It is evident in the present context that the expressions with DIY (e.g., “DIY cat bed” in response to *box*) and without DIY (“cat bed”) mean the same usage of the object. Because we noted that the inclusion of DIY increases the semantic distance scores produced by SemDis, we removed the DIYs from the responses before entering them into the analysis. For the same reason, we removed also expressions “Make a _____”, Making a _____, Use as a ___ “ from the beginning of the responses.

For each response in AUT tasks, semantic distance between the object name and the response was computed with five semantic models and their mean value was used in further processing (for detailed description of the models, see^23^). For statistical analyses, we computed for each participant and for each AI test session both the mean semantic distance score across all the responses generated to each probe object during a session, and the maximum score from the responses to each object (i.e., the highest score from all the responses to an object during a session). In the statistical analyses, each AI session was processed as it were from an individual participant; therefore, we got 11 observations per object for each chatbot.

We collected subjective creativity/originality ratings from six briefly trained humans. They were not told that some of the responses were generated by AI. They rated each response for creativity/originality using 5-point likert scale (1 = *not at all*, 5 = *very*). The instruction stressed that they should stress novelty over usefulness and use the instruction given for participants as the reference point against which to evaluate the responses. They were explicitly instructed that a common use, such as “cutting” in response object *scissors*, should be given a low score, and that also a confusing or illogical response as well as a lacking response should receive score 1. Each rater had a different order in which the four objects were evaluated. The order in which the responses within object categories were presented was randomized separately for each rater. The scores of each rater were averaged across all the responses a participant (or chatbot in a session) gave to an object, and the final subjective scores for each object were formed by averaging the 6 raters’ scores, The inter-rater reliability was assessed by calculating Intraclass Correlation Coefficients (ICCs; model = "twoway", type = "consistency", unit = "average") with irr package (https://CRAN.R-project.org/package=irr). In this model systematic differences between raters were irrelevant. The ICCs were 0.88, 95% CI [0.86, 0.90] for rope, 0.93, 95% CI [91, 94] for box, 0.90, 95% CI [0.88, 0.82] for pencil, and 0.93, 95% CI [0.92, 0.94] for candle.

### Statistical analyses

Separate linear mixed-effect analyses were performed with lme4 package^24^ and lmerTest^25^ in R^26^ on the mean scores and max scores, both for the semantic distance scoring and humans’ subjective scoring. In the first set of models, the only fixed effects were Group (human vs. ai) and Fluency (i.e., the count of responses), and random intercept for participants (and session for AI) served as the random effect. In the next set of analyses, Group and Object, and their interactions were the fixed effects, and Fluency (i.e., number of responses) served as the covariate. The group variable consisted of four levels (human, ChatCPT3, Chat GPT4, Copy.Ai) and the object variable involved four levels (rope, box, pencil, candle). In these analyses the R’s anova function was applied on the models to obtain Type III analysis of variance results (Satterthwaite's method) as it makes the interpretation of main effects and interactions simpler than the standard outputs of the linear mixed-effect models. The post-hoc pairwise comparisons were adjusted for multiple comparison with mvt method in package emmeans v.1.8.2. (https://CRAN.R-project.org/package=emmeans). For simplicity, we refer to 95% CI as CI in the results section.

## Results

### Descriptive statistics and correlations

**Figure 1:**
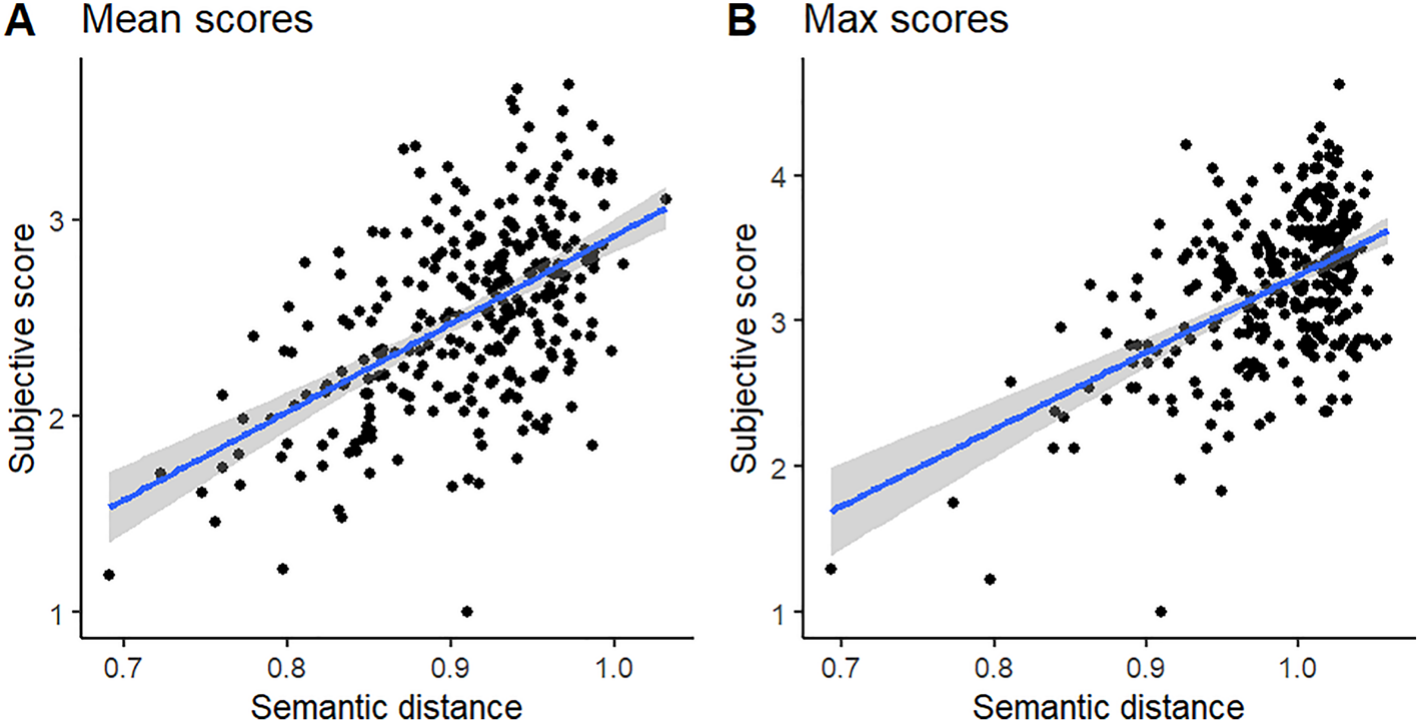
The relationship between the semantic distance scores of originality and the human-made subjective ratings for (**A**) the mean scores and (**B**) the max scores.

Table 1 shows the descriptive statistics for humans and AI chatbots, averaged across all the four object prompts. The correlation between the semantic distance and humans’ subjective ratings in the mean scores was 0.55, 95% CI [0.46, 0.62], p < 0.001 (Fig. 1A). The corresponding correlation between max scores was 0.52, 95% CI [0.43, 0.60], p < 0.001 (Fig. 1B). The correlations were moderate, suggesting that these two scoring methods measure similar both not identical attributes of creative divergent thinking. Thus, it is reasonable to analyze the data separately for semantic distance and subjective ratings.

**Table 1:** Descriptive statistics for the mean and max semantic distance and subjective ratings of creativity for artificial intelligence (AI) and human participants averaged across all responses to the object probes.

	Group	Mean semantic distance	Max semantic distance	Mean subjective rating	Max subjective rating
N^a^	AI	33	33	33	33
	Human	256	256	256	256
Mean	AI	0.95	1.01	2.91	3.61
	Human	0.91	0.98	2.47	3.18
95% CI	AI	[0.94, 0.96]	[1.00, 1.02]	[2.77, 3.05]	[3.45, 3.77]
	Human	[0.90, 0.91]	[0.97, 0.99]	[2.41, 2.53]	[3.12, 3.25]
SD	AI	0.03	0.036	0.39	0.45
	Human	0.06	0.053	0.47	0.53
Min	AI	0.90	0.90	2.21	2.71
	Human	0.69	0.69	1.00	1.00
Max	AI	1.01	1.04	3.69	4.63
	Human	1.03	1.06	3.56	4.25

Theoretically the semantic distance may vary between 0 and 2, with higher scores indicating higher distance. The subjective ratings of creativity were made on 5-point scale (1 = *not at all*, 5 = *very*).

^a^N for AI refers to the number of test sessions.

### Overall human and AI performance

**Figure 2:**
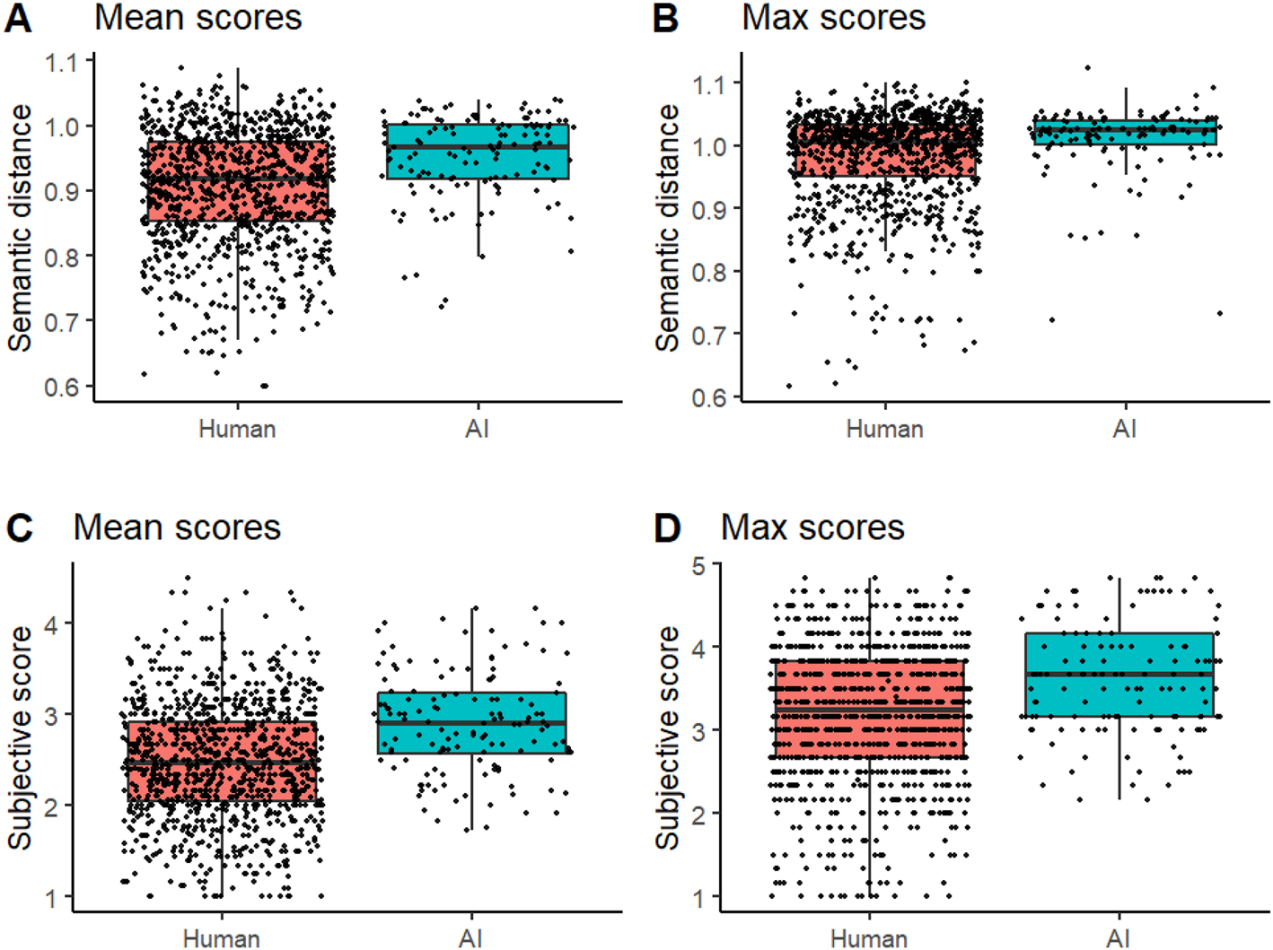
Humans’ and AI’s mean scores (average of all responses within each trial) and max scores (the highest scoring response within each trial) as revealed by sematic distance analysis (**A**, **B**) and human subjective ratings (**C**, **D**).

To get an overall picture of the differences between humans and AI, we began the analyses with linear mixed-effect models with Group (human, AI) as the fixed effect and Fluency as a covariate. Figure 2 presents the results combined across AI chatbots and objects. Overall, AI’s scores were higher than humans’ ones. The semantic distance mean scores (Fig. 2A) and max scores (Fig. 1B) were higher for AI than humans, *B* = 0.049, *SE* = 0.010, *CI* [0.030, 0.069], *t*(274) = 4.949, *p* < 0.001 and *B* = 0.027, *SE* = 0.009, *CI* [0.010, 0.044], *t*(268) = 3.037, p = 0.003, respectively. Fluency as a covariate decreased the mean scores, *B* = −0.012, SE = 0.003, *CI* [−0.017, −0.006], *t*(279) = −4.170, *p* < 0.001, and increased the max scores, *B* = 0.011, *SE* = 0.002, *CI* [0.006, 0.016], *t*(274) = 4.486, *p* < 0.001.

The human subjective ratings of creativity showed similar results. The mean scores (Fig. 2C) were higher for AI than humans, *B* = 0.453, *SE* = 0.082, *CI* [0.292, 0.614], *t*(282) = 5.496, *p* < 0.001, and fluency decreased the mean scores, *B* = −0.088, *SE* = 0.023, *CI* [−0.133, −0.044], *t*(284) = −3.877, *p* < 0.001. Additionally, the max scores were higher for AI than humans (Fig. 2D), *B* = 0.403, *SE* = 0.091, *CI* [0.226, 0.581], *t*(280) = 4.444, *p* < 0.001. Fluency increased the max scores, *B* = 0.139, *SE* = 0.025, *CI* [0.090, 0.188], *t*(282) = 5.533, *p* < 0.001.

The distribution of the subjective scores in Fig. 2C and 2D shows that there were several humans’ observations whose mean and max scores were between 1 and 2, implying that they responded either with the typical uses of the object or gave an illogical or confused response. By contrast, the AI chatbots scored systematically higher than humans in the lower span of the scale, and their subjective max scores were never below 2, suggesting that the chatbots responded predominantly with unusual and logical uses. The lack of very low scores of the AI chatbots is observable also in the semantic distance scores (Fig. 2A and B), but the continuous numerical scale is not interpretable in similar way as the subjective scores (see Scoring section).

### Differentiating performance between AI chatbots and objects: semantic distance

Next, we studied in more detail the responses of humans and each AI chatbot to each object, with Group (human, ChatGPT3, ChatGPT4, Copy.Ai) and Object (rope, box, pencil, candle) and their interactions as fixed effects and Fluency as a covariate. The effect of Fluency was statistically significant in all the following analyses, showing similar pattern as in previous analyses (decreasing mean scores and increasing max scores), so we do report them.

**Figure 3:**
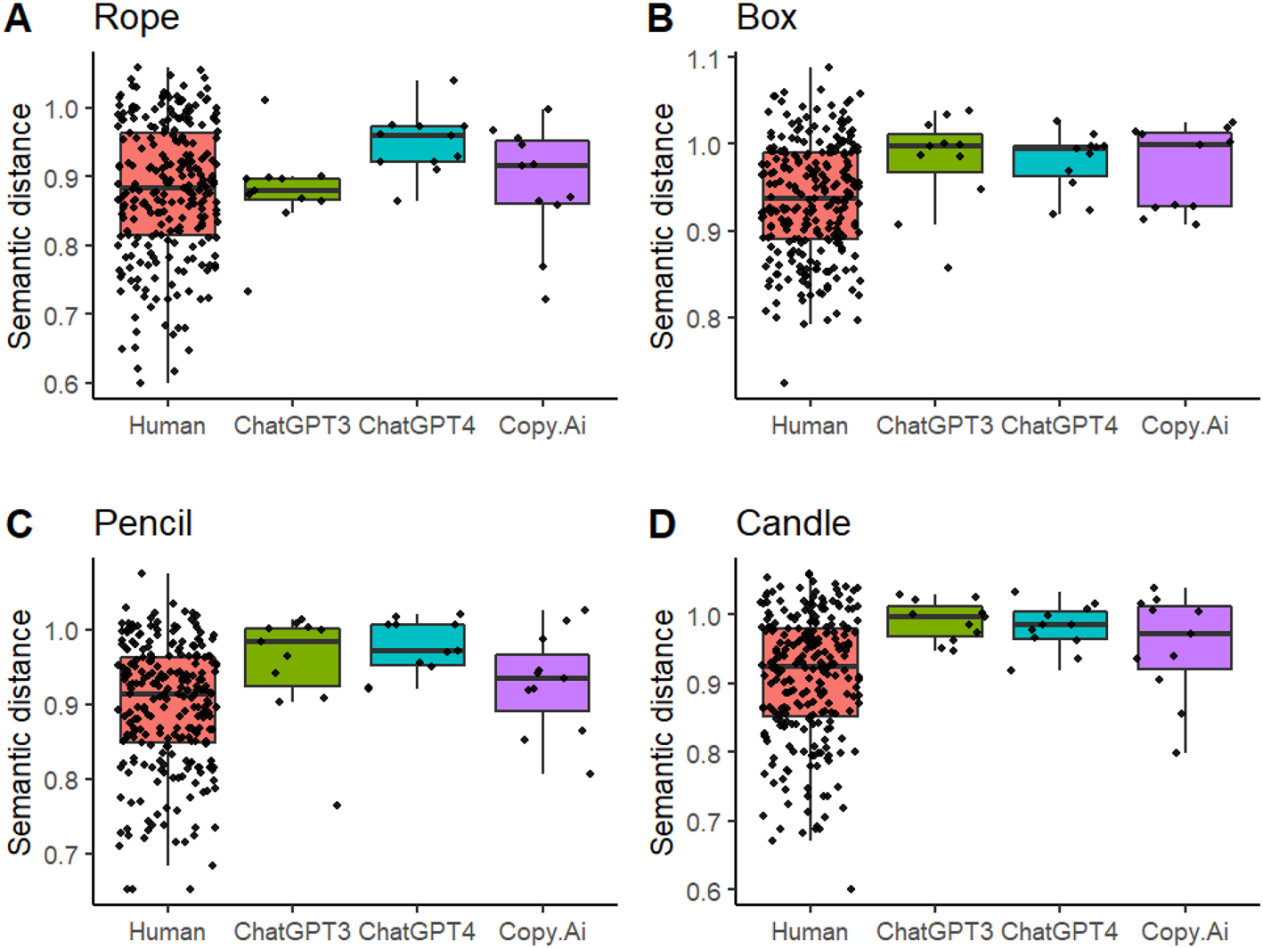
Mean semantic distance scores of the humans and chatbots to the four objects.

The analysis of mean semantic distance (Fig. 3) showed a main effect for Group, *F*(3, 273) = 9.000, *p* < 0.001. Post-hoc pairwise comparisons with ‘mvt’ adjustment for multiple comparisons indicated that this effect was due to ChatGPT3, *t*(282) = −2.867, *CI* [−0.090, −0.005], *p* < 0.0213, and Chat GPT4, *t*(282) = −4.115, *CI* [−0.110, −0.026], *p* < 0.001, obtaining higher mean semantic distance scores than humans. Semantic distance differed between the objects, *F*(3, 836) = 10.102, *p* < 0.001, with responses to rope receiving lower scores than those to box, *t*(845) = −5.030, *CI* [−0.102, −0.033], *p* < 0.001, pencil, *t*(845) = −2.997, *CI* [−0.075, −0.007], p = 0.015, and candle, *t*(845) = −4.445, *CI* [−0.094, −0.025], *p* < 0.001. The interaction between Group and Object was not statistically significant, *F*(9, 836) = 1.098,* p* = 0.361.

**Figure 4:**
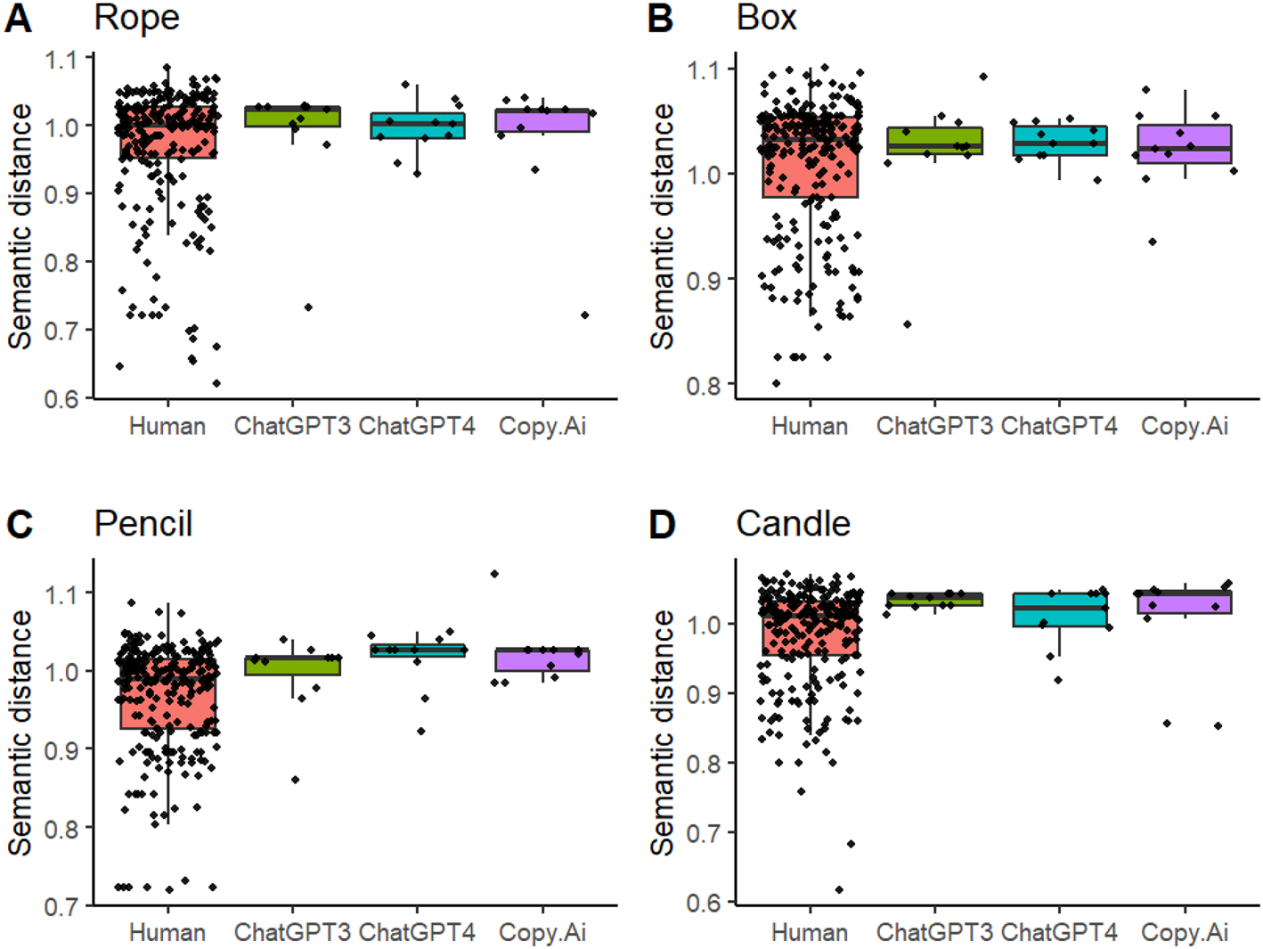
Max semantic distance scores of the humans and the chatbots to the four objects.

The analysis of max semantic distance (Fig. 4) also showed a main effect for Group, *F*(3, 266) = 3.088, *p* = 0.028, but post-hoc pairwise comparisons did not reveal any statistically significant differences between the groups (human, ChatGPT3, ChatGPT4, Copy.AI) after accounting for multiple comparisons (all *p*-values > 0.223). The main effect for object, *F*(3, 825) = 3.256, *p* = 0.021) resulted from the responses to *box* receiving higher scores than those to *rope*, *t*(839) = −3.055, *CI* [−0.067, −0.006], p = 0.0124. Group and object did not interact statistically significantly, *F*(9, 825) = 0.641, *p* = 0.762.

In summary, mean semantic distance scores of ChatGPT3 and ChatGpt4 were higher than those of humans, but no statistically significant differences between the AI chatbots were detected. However, it can be noted from Fig. 3 that none of the chatbots’ mean scores were higher than the highest score of humans. The max scores did not reveal any statistically significant differences between the humans and the AI chatbots. Figure 4C reveals, however, that only one max score of the AI chatbots, Copy.Ai’s max score to *pencil* (1.124), was higher than the corresponding highest human max score (1.101).

### Differentiating performance between AI chatbots and objects: human subjective ratings

**Figure 5:**
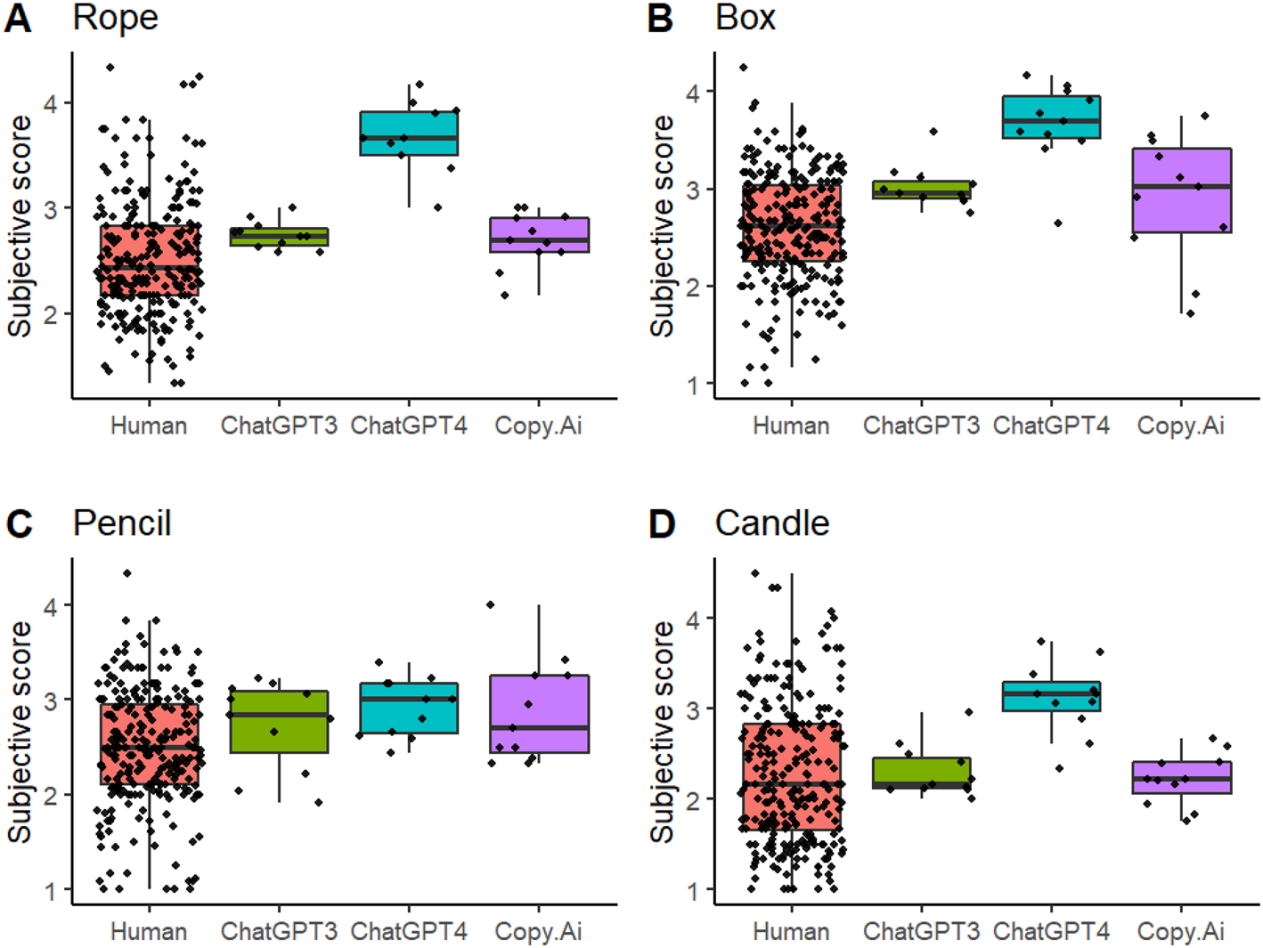
Mean scores based on subjective ratings for the humans and chatbots to the four objects.

The analysis of human subjective rating mean scores (Fig. 5) revealed the main effects of Group, *F*(3, 280) = 16.147,* p* < 0.001, and Object, *F*(3, 847) = 14.920, *p* < 0.001. The performance of ChatGPT4 was superior: its responses received on average higher points than humans, *t*(283) = −6.6649, *CI* [−1.23, −0.547], *p* < 0.001, ChatGPT3, *t*(283) = −3.459, *CI* [−1.112 −0.1674], *p* = 0.003, and Copy.AI, *t*(283) = 3.609, *CI* [0.195, 1.139], *p* = 0.002, which did not differ between each other. However, the superiority of ChatGPT4 could not be generalized to object *pencil*, as suggested by the Group × Object interaction, *F*(9, 849) = 2.486, *p* = 0.008. Responses to *candle* received lower ratings than responses to *rope*, *t*(852) = 4.788, *CI* [0.198, 0.6587], *p* < 0.001, box, *t*(852) = 3.283, *p* = 0.006, *CI* [0.341, 0.802], and *pencil*, *t*(852) = 3.104, *CI* [0.047, 0.508], p = 0.011.

**Figure 6:**
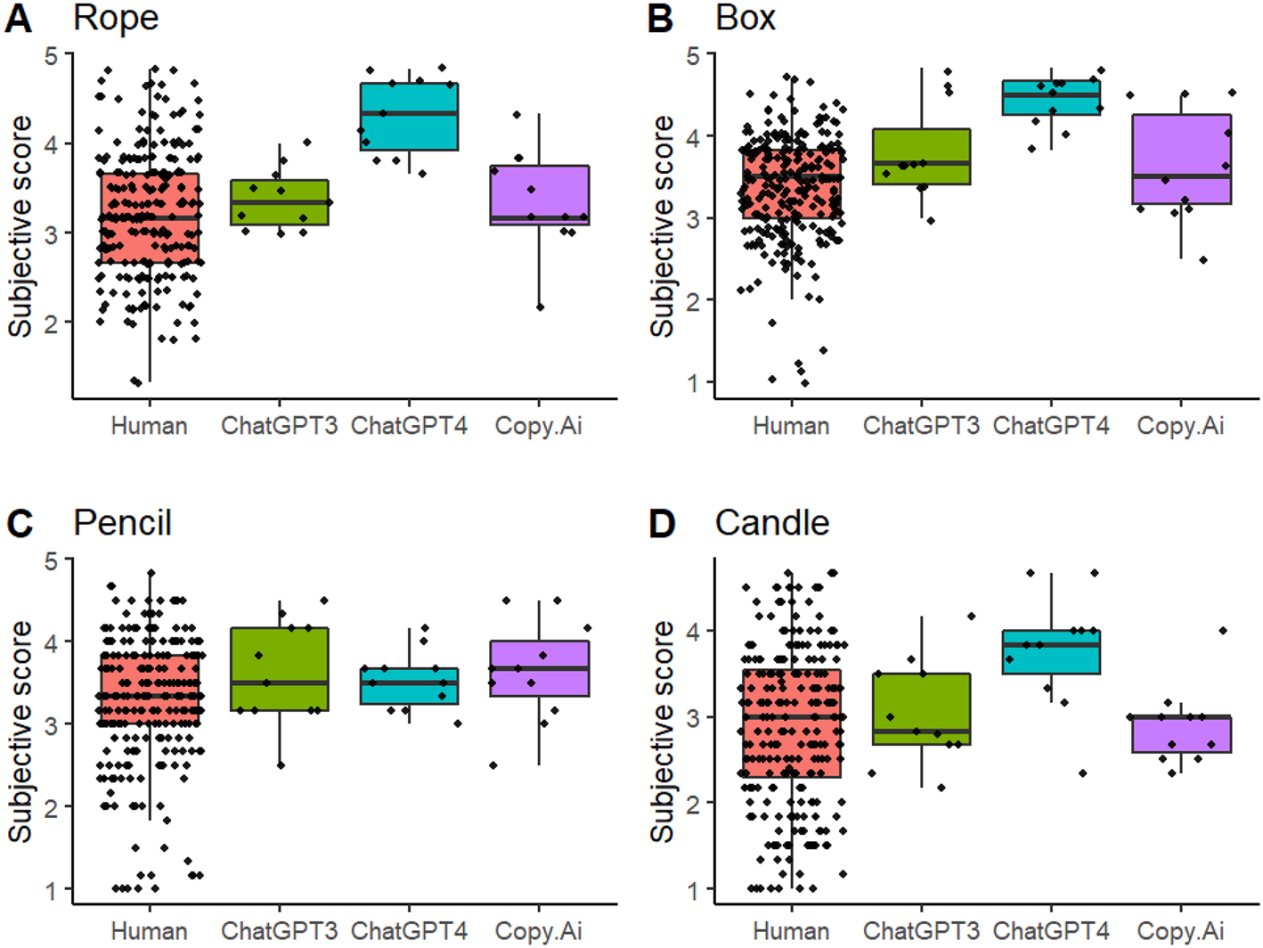
Max scores based on subjective ratings for the humans and chatbots to the four objects.

Figure 5 suggests that the observed pattern of results for subjective rating max scores was similar as that for the corresponding mean scores. The main effect for Group, *F*(3, 278) = 10.612, *p* < 0.001) was due to ChatGPT4 getting higher scores than humans, *t*(283) = −5.400, *CI* [−1.182, −0.423], *p* < 0.001, ChatGPT3, *t*(283) = −2.711, *CI* [−1.083, −0.032], *p* = 0.033, and Copy.Ai, *t*(283) = 3221, *CI* [0.114, 1.166], *p* = 0.010. Although the boxplots in Fig. 6C suggest that on average ChatGPT4 performed similarly as the other chatbots in response to *pencil* and lower as compared with its own responses to the other objects, the Group × Object interaction did not reach statistical significance, *F*(9, 845) = 1.801, *p* = 0.064. Similarly to subjective mean scores, the subjective max scores in response to *candle* were lower than those to *rope*, *t*(849) = 3.561, *CI* [0.116, 0.721], *p* = 0.002, box, *t*(849) = 5.541, *CI* [0.349, 0.953], *p* < 0.001, and *pencil*, *t*(849) = 3.126, *CI* [0.065, 0.669], *p* = 0.010. There were two AI sessions where the max score in response to *box* was higher than the corresponding highest human max score 4.67 (Fig. 6B). They were ChatGPT3’s and ChatGPT4’s max responses to *box* (4.83, for both chatbots).

## Discussion

We compared the performance of AI chatbots and human participants in a typical divergent thinking task, AUT. On average, the AI chatbots outperformed the human participants in both mean scores (all responses to an object averaged) and max scores (the best response to an object). This advantage was observed for both the semantic distance of the responses and the subjective ratings of creativity provided by unbiased human raters who were unaware of that some of the responses were generated by AI.

The standard definition describes creativity as the ability to produce ideas that are, to some extent, original and useful^12^. This definition does not specify the internal processes that produce the creative idea, but instead attribute creative ability to an agent based on the creative products.

Therefore, the present empirical data shows that AI can produce creative outputs that have reached at least the same level, and even higher, as the average of humans in this task. Just as in the case of the arts^8^, these results suggest that the production of creative ideas may not be a feature only displayed in conscious human beings.

Although AI chatbots performed better than humans on average, they did not consistently outperform the best human performers. There was only one instance in which an AI chatbot achieved the highest semantic distance score (Copy.Ai in response to *pencil*) and two instances where AI chatbots (ChatGPT3 and ChatGPT4 in response to *box*) achieved the highest subjective scores. In all other cases, the highest scores were achieved by humans. However, it is evident from Figs. 2, 3, 4, 5 and 6 that humans consistently achieved the lowest scores in the tasks. While AI chatbots typically responded with relatively high levels of creativity and some variability, human performance exhibited greater variation, as measured by both semantic distance and subjective ratings.

What explains the differences between humans and AI? Subjective ratings of creativity are more revealing than semantic distance scores, as raters were explicitly asked to give low scores to common or illogical uses. In 7% of trials, humans received max ratings lower than 2, while AI never received such low ratings. This suggests that, instead of generating new ideas, humans were overrepresented in producing common or low-quality responses. Based on the controlled-attention theories of creativity^21,22^, it seems that the weakness in human creative thinking, compared to AI chatbots, lies in executive functions. There are several ways in which human executive functions may have failed. For example, humans may have had difficulties in maintaining task goals in working memory, failures in inhibiting the activation of close concepts, and in switching attention to distantly related concepts^21,22^. In addition, the contribution of motivational^27^ and affective^28^ factors on human performance cannot be ruled out, while we may assume that the AI chatbots executed the task always at the best of their capabilities from a computational point of view.

However, the overall superiority of AI cannot be solely explained by the low executive performance in some humans, as the AI's superiority is still evident even when the lowest performing humans are not considered (see additional analyses in Supplementary Materials). In associative theories of creativity, individuals differ in their structure of semantic memory and creativity is linked to flexible and highly connected semantic networks^18,19^. It is not exactly clear how semantic networks are represented in the memory of current AI chatbots, but their speed in accessing large data structures, whether flexible or not, may explain their higher-level average performance compared to the average human performance. However, the semantic distance scores of the AI chatbots' best responses were not always systematically larger than those of the best humans. Thus, highly creative individuals, who likely possess flexible semantic networks, can still compete with AI in activating distant, weakly related concepts.

One question that arises from the results is why ChatGPT4, the newest and most efficient AI chatbot currently available, performed so well according to human raters, compared to humans and other AI chatbots. According to OpenAI, ChatGPT4 can process eight times more words at once than ChatGPT3. However, ChatGPT4 was not better than the other chatbots as measured with the “objective” semantic distance. This suggests that access to remote concepts alone may not explain why ChatGPT4's responses were evaluated as so creative. Perhaps an explanation lies in a more nuanced and surprising way the concepts were combined by ChatGPT4. For example, in one session, ChatGPT4 responded to *box* with “*cat amusement park*,” while one human and ChatGPT3 responded with “*cat playhouse*,” which received lower creativity ratings. The correlations between semantic distance and humans' subjective ratings were relevant (> 0.50), but far from perfect, suggesting that they measured only partially the same aspects of creativity. Human raters may be more sensitive than automatic algorithms to recognize surprise or other emotional components in the combined concepts, and ChatGPT4 seems to be able to include such components in its ideas.

A limitation of in our study is the restricted number of observations from each chatbot, which limited the statistical power, especially in comparisons between individual chatbots. Additionally, to control for the confounding effects of fluency and elaboration^29^, we had to ask the chatbots to produce specific amounts of responses and limit the word count in their responses, even though they are capable of generating several ideas within seconds. This purposely impaired the potential of the AI. Comparing human and chatbot creativity at process levels seems impossible, because chatbots are “black boxes”, and we cannot know precisely how they generate responses or what information they have access to. It remains possible that they simply retrieve ideas that exist in their database. In such a case, their performance would merely reflect semantic retrieval, not creativity in the sense of combining concepts in new ways. The same problem exists with human participants who may retrieve ideas they have encountered previously. Future studies should develop completely new tests for which no prior ideas exist. Moreover, the human group consisted of young and middle-aged adults from Western countries, which limits generalizations of the differences between humans and AI.

Understanding how AI systems and humans interpret, understand, and articulate language could potentially bridge the gap between machine efficiency and human intuition. As we move forward, it becomes imperative for future research to explore avenues where AI can be integrated to bolster and amplify human creativity, thereby fostering a close interaction between technology and human potential.

## Conclusions

The study provides insights into the relationship between human and machine creativity. The results suggest that AI has reached at least the same level, or even surpassed, the average human's ability to generate ideas in the most typical test of creative thinking (AUT). Although AI chatbots on average outperform humans, the best humans can still compete with them. However, the AI technology is rapidly developing and the results may be different after half year. On basis of the present study, the clearest weakness in humans' performance lies in the relatively high proportion of poor-quality ideas, which were absent in chatbots' responses. This weakness may be due to normal variations in human performance, including failures in associative and executive processes, as well as motivational factors. It should be noted that creativity is a multifaceted phenomenon, and we have focused here only on performance in the most used task (AUT) measuring divergent thinking.

### Supplementary Information

Supplementary Information.

## Data Availability

The data matrix and analysis scripts are available at OSF.io (https://osf.io/qdz3n/?view_only=d2da32d06f0f4f7ca5bd4bdfc8cf4346).
