# Author Correction: Best humans still outperform artificial intelligence in a creative divergent thinking task

**DOI:** 10.1038/s41598-024-54745-y

**Published:** 2024-02-20

**Authors:** Mika Koivisto, Simone Grassini

**Affiliations:** 1 Department of Psychology, University of Turku, Turku, Finland; 2 Department of Psychosocial Science, University of Bergen, Bergen, Norway; 3 Cognitive and Behavioral Neuroscience Laboratory, University of Stavanger, Stavanger, Norway

Correction to: *Scientific Reports* 10.1038/s41598-023-40858-3, published online 14 September 2023

The original version of this Article contained errors in the Results, under the subheading ‘Overall human and AI performance’, where the data analysis related to Figure 2D was incorrect due to an error in the data analysis code.

As a result,

“Additionally, the max scores were higher for AI than humans (Fig. 2D), *B* = 0.453, *SE* = 0.082, *CI* [0.286, 0.613], *t(*282) = 5.495, *p* < 0.001. Fluency decreased the max scores, *B* = −0.088, *SE*= 0.023, *CI* [−0.133, −0.043], *t*(283) = −3.877,* p* < 0.001.”

now reads:

“Additionally, the max scores were higher for AI than humans (Fig. 2D), *B* = 0.403, *SE* = 0.091, *CI* [0.226, 0.581], *t*(280) = 4.444, *p* < 0.001. Fluency increased the max scores, *B* = 0.139, *SE*= 0.025, *CI* [0.090, 0.188], *t*(282) = 5.533, *p* < 0.001.”

The original Article has been corrected.

