# Supplementary material for: Best humans still outperform artificial intelligence in a creative divergent thinking task
Source: Sci Rep. 2023 Sep 14;13:13601. doi: 10.1038/s41598-023-40858-3 (PMC10502005; doi:10.1038/s41598-023-40858-3)
Supplement: Supplementary file 1 — Supplementary Information. [file 41598_2023_40858_MOESM1_ESM.docx]

**Best humans still outperform artificial intelligence in a creative divergent thinking task**

**Mika Koivisto & Simone Grassini**

**Supplementary Materials**


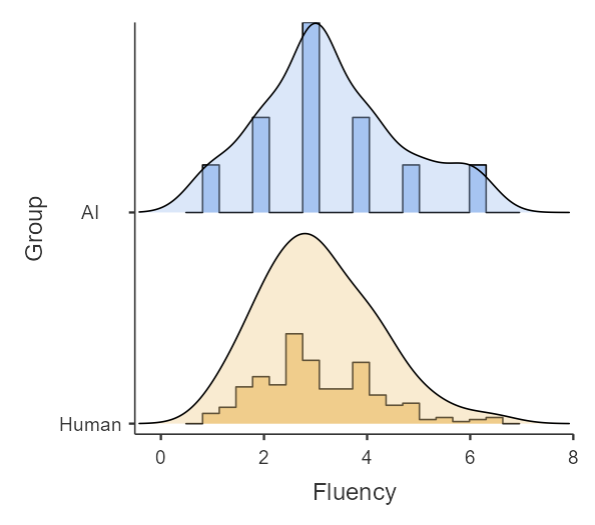


**Supplementary Figure 1.** Histograms and density plots of fluency (the number of responses) for AI chatbots and humans.

**Supplementary models**

These models included Group (Human vs. AI) and fluency as fixed effects and random intercept for participants (session for AI) as a random effect.

*Subjective mean scores* (observations, *n* = 896; participants/sessions, *n* = 280): When observations with lower than 2 points in subjective mean ratings were eliminated, AI still performed better than humans, *B* = 0.260, *t*(236) = 4.738, *CI* [0.152, 0.367], *p* < .001. Fluency was negatively related to subjective mean scores, *B* = -0.108, *t*(269) = 6.671, *CI* [-0.139, -0.076], *p* < .001.

*Subjective max scores* (observations, *n* = 1057; participants/sessions, *n* = 286): When observations with lower than 2 points in subjective max ratings were eliminated, AI still performed better than humans, *B* = 0.259, *t*(262) = 3.764, *CI* [0.124, 0.393], *p* < 0.001. Fluency was positively related to subjective max scores, *B* = 0.085, *t*(280) = 4.293, *CI* [0.046, 0.123], *p* < .001.
